# Electrospinning of Quaternized Chitosan-Poly(vinyl alcohol) Composite Nanofiber Membrane: Processing Optimization and Antibacterial Efficacy

**DOI:** 10.3390/membranes12030332

**Published:** 2022-03-17

**Authors:** Jheng-Yu Wu, Chi-Yun Wang, Kuei-Hsiang Chen, You-Ren Lai, Chen-Yaw Chiu, Hung-Che Lee, Yu-Kaung Chang

**Affiliations:** 1 Department of Chemical Engineering, Ming Chi University of Technology, New Taipei City 243303, Taiwan; m05138207@mail2.mcut.edu.tw (J.-Y.W.); khchen@mail.mcut.edu.tw (K.-H.C.); chenyaw@gmail.com (C.-Y.C.); 2 International Ph.D. Program in Innovative Technology of Biomedical Engineering and Medical Devices, Ming Chi University of Technology, New Taipei City 243303, Taiwan; chiyunw@mail.mcut.edu.tw; 3 Department of Chemical Engineering, National Taiwan University, Taipei 10617, Taiwan; ray110135@gmail.com; 4 Falco Tech Enterprise Co., Ltd., Tucheng Dist., New Taipei City 23674, Taiwan; falco.tec@msa.hinet.net

**Keywords:** nanofiber membrane, chitosan, HTCC, polyvinyl alcohol, antibacterial, *Escherichia coli*

## Abstract

*N*-(2-hydroxy) propyl-3-trimethylammonium chitosan chloride (HTCC) is a type of quaternary ammonium chitosan derivative with an antibacterial activity superior to the pristine chitosan, but its electrospinnability is limited. In this study, polyvinyl alcohol (PVA) was blended with HTCC to improve the electrospinnability of nanofibers. The electrospinning of PVA–HTCC nanofiber membranes was optimized in terms of structural stability and antimicrobial performance. Based on scanning electron microscopic analysis, the morphology and diameter of the produced nanofibers were influenced by the applied voltage, flow rate of the feed solution, and weight ratio of the polymer blend. An increase in the HTCC content decreased the average nanofiber diameter. The maximum water solubility of the PVA–HTCC nanofibers reached the maximum value of 70.92% at 12 h and 25 °C. The antibacterial activity of PVA–HTCC nanofiber membranes against *Escherichia coli* was ~90%, which is significantly higher than that of PVA–chitosan nanofiber membrane. Moreover, the antibacterial efficiency of PVA–HTCC nanofiber membranes remained unaffected after 5 cycles of antibacterial treatment. The good antibacterial performance and biocompatibility of PVA–HTCC nanofiber membrane makes them attractive for biomedical and biochemical applications that necessitate sterile conditions.

## 1. Introduction 

Electrospinning is a popular, low-cost, versatile, and straightforward technique for fabricating nanoscale fibers. Commonly, electrospinning is applied in various fields, including tissue engineering, water treatment, energy generation, and environmental remediation [1,2,3,4,5]. In particular, electrospinning can serve as an alternative technique to the well-established conventional phase-inversion method for the preparation of nanofiber membranes with highly porous structures [2,3,6]. Today, electrospinning technique has been regarded as a mature technology, where there is a wide range of materials electrospinnable for different commercial applications. Dedicated companies (e.g., Elmarco, Ltd., Liberec, Czech Republic) supply laboratory and industrial-scale components and apparatus for electrospinning [7].

Electrospun nanofiber membranes can be functionalized prior to the electrospinning (i.e., incorporation of functional additives in polymer blends) or after the fabrication process (i.e., surface modification of nanofiber membranes by a post-treatment method) [8]. Surface modification of nanofiber membranes can be performed by either physical adsorption or multi-step chemical grafting reactions [5,9]. The blending of flexible polymer additives before electrospinning is relatively straightforward [10,11,12] and feasible for large-scale production. Nevertheless, the optimal operating conditions of electrospinning are essential for the successful preparation of electrospun nanofibers. 

Natural biopolymers have been widely used as materials for nanofibers due to their unique biological features, including non-toxicity, biodegradability, and biocompatibility [10]. A good example in the class of naturally occurring polysaccharide is chitosan, which contains (1–4) acetamido 2 deoxy-*β*-D glucan residues and is derived from the deacetylation of chitin. Chitosan exhibits cationic characteristics and antimicrobial properties [11,12,13]. Electrospinning of chitosan into fiber structure can be achieved by incorporating flexible polymers with functional additives [e.g., polyethylene oxide or polyvinyl alcohol (PVA)] into the chitosan solution [14,15,16,17,18,19,20,21,22]. This is because of the poor water solubility and low antimicrobial efficacy of chitosan at high pH [23,24].

*N*-(2-hydroxy) propyl-3-trimethylammonium chitosan chloride (HTCC) can be produced from the quaternary ammonium chitosan derivatives, such as glycidyl trimethyl ammonium chloride (GTMAC). Although HTCC has a higher antibacterial efficacy and water solubility than chitosan, the low viscosity of a pure HTCC aqueous solution makes it non-electrospinnable [25]. Nevertheless, the problem can be alleviated by the addition of PVA [16,23,24]. PVA is a synthetic polymer widely used in the biomedicine-relevant fields [26,27,28] owing to its non-toxic nature, high water solubility, good biocompatibility, and biodegradability. There are many published articles related to PVA–chitosan nanofibers and PVA–HTCC nanofibers [24,25,29,30,31,32,33,34,35]. Some examples of the applications of these chitosan- and HTCC-related materials used in the antibacterial process are described as below:

Mi and Heldt reported that the PVA–HTCC nanofibers have potential as a filter material for the removal of pathogens from drinking water [30]. Wang et al. reported that the antimicrobial activity of the PVA–HTCC nanofiber membrane was better than that of the PVA–chitosan nanofiber membrane. When the mass ratio of PVA–HTCC = 6:4, the antibacterial rates of the membrane against *Escherichia coli* and *Staphylococcus aureus* were over 99% [31]. Bai et al. reported that the HTCC–graphene composite nanofibers could be incorporated into microfiltration membranes and effectively remove viruses by adsorption. This would create a low-pressure system that was more likely to benefit areas in need of freshwater [32]. Moreover, Hu and Wang reported that a blend membrane of HTCC and PVA prepared by simple mixing and casting method showed a high antibacterial activity against *S. aureus* and *E. coli* [33]. Lim and Hudson reported that the fiber-reactive chitosan derivative, O-acrylamidomethyl-HTCC (NMA–HTCC), showed the antimicrobial activity of the NMA–HTCC was effective against *S. aureus* and *E. coli* [34]. Cheah et al. reported that the electrospinning PAN nanofiber membrane (P-CN) was hydrolyzed to convert carboxylic groups (P-COOH) and covalently graft chitosan molecules. The chitosan modified membrane (P-CS), can be functionalized with quaternary amine (i.e., glycidyl trimethyl ammonium chloride, GTMAC) to form quaternized chitosan nanofiber membrane (designated as P-HTCC) under various conditions (acidic, neutral, and alkaline); The antibacterial activity (against *E. coli*) of the P-HTCC nanofiber membrane (modified using an acidic medium) was 99.95%. In our recent study [29], the PVA–HTCC nanofiber membrane was crosslinked with the blocked diisocyanate (BI) to enhance the stability of the nanofiber membrane in water; the past study only focused on the synthesis of a non-water-soluble PVA–HTCC-BI nanofiber membrane for antibacterial application. The antibacterial efficacy of the PVA–HTCC–BI composite nanofiber membrane was mainly investigated by the amount of BI added and the hot-pressing conditions of the membrane. However, these articles did not explore the optimal electrospinning conditions for PVA–HTCC nanofiber in detail [24,25,29,30,31,32,33,34,35]. There are no systematic investigations on the electrospinning conditions for these kinds of composite nanofibers, such as the mass ratio of the materials, applied voltage, and extrusion rate. Moreover, the water solubility and biocompatibility of the PVA–HTCC composite nanofiber membranes were not investigated. 

This work aimed to evaluate the processing conditions of electrospinning on the structural stability and antimicrobial efficacy of the composite PVA–HTCC nanofibers without BI. The effects of operating parameters on the average fiber diameters and morphology of PVA–HTCC nanofiber membranes were investigated; these parameters included the applied voltage, flowrate of spinning solution, and weight ratio of PVA to HTCC. The PVA–HTCC nanofiber membranes were analyzed using a scanning electron microscope (SEM) and Fourier-transform infrared (FTIR) spectroscopy. In this work, we optimized the electrospinning for PVA–chitosan and PVA–HTCC nanofiber membranes. The water solubility and cytotoxicity of PVA-HTCC nanofiber membrane were further extensively investigated. Finally, the antibacterial potency of the PVA–HTCC nanofiber membrane against *Escherichia coli* (*E. coli*) was also examined and compared with PVA–chitosan nanofiber membranes. The feasibility of reusing the PVA–HTCC nanofiber membranes was also tested.

## 2. Materials and Methods

### 2.1. Materials 

Polyethylene terephthalate (PET) spunbond fabric (basis mass: 15 g·m^−2^, thickness: 90 μm, fibre diameter: 300–500 μm) was acquired from Freudenberg Far Eastern Spunweb Co., Ltd. (Taoyuan, Taiwan). PVA (Mw: 75,000 to 180,000 g·mol^−1^) and chitosans (average *Mw*: 340 kD; deacetylation degree, DD: 90–95%) were obtained from Charming & Beauty Co., Ltd. (Taipei, Taiwan). GTMAC and all other unmentioned chemicals used in the experiments were purchased from Sigma-Aldrich (St. Louis, MO, USA). 

### 2.2. Preparation and Physical Characteristics of Nanofiber Membranes

**Figure 1:**
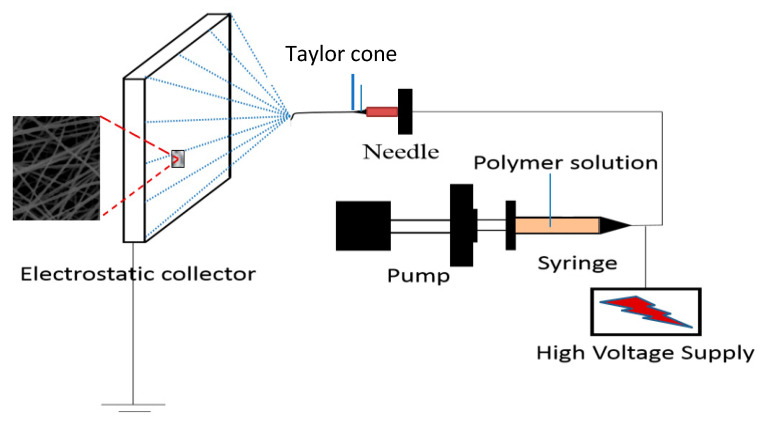
Schematic representation of the electrospinning device containing syringe needle, syringe pump, high-voltage direct current supply, and collector.

The electrospinning device used in this study was supplied by Falco Tech Enterprise Co., Ltd. (Taoyuan, Taiwan). The assembly and operation of the electrospinning device was in accordance with the procedures described in a previous study [20,29], and the setup of the device is illustrated in Figure 1. During electrospinning, the humidity was approximately 65–70%. The electrospinning process was performed at 25 °C by ejecting the polymer solution via a nozzle tip to an electrostatic collector pre-immobilized with PET, which serves as a supporting layer of nanofiber membrane. Lastly, the heat pressing of the electrospun nanofiber membranes was conducted at 100 °C for 1 h and the pressure used for the heat pressing was 1 MPa. The morphology of the nanofiber membrane was examined using a SEM (Hitachi, Model S-2600H/EDX, Tokyo, Japan). Before SEM examination, all samples were sputter-coated with platinum. The chemical properties of nanofiber membranes were characterized using a FTIR spectrometer (Perkin Elmer, Model Spectrum One, NY, USA) with a resolution of 1 cm^−1^ in a wavenumber range of 4000–400 cm^−1^. 

#### 2.2.1. Preparation of PVA–Chitosan Nanofiber Membrane

A 10% (*w*/*w*) PVA solution was prepared by dissolving the PVA in distilled water at 80 °C under stirring for 8 h. Chitosan (average *Mw*: 34 kD) was dissolved in acetic acid solution (1%) for preparation of 3% (*w*/*w*) chitosan stock solution. Both solutions were subsequently mixed at different ratios of PVA to chitosan. The ranges of variables tested in the experiments were listed as follows: PVA:chitosan ratio (90/10–50/50), applied voltage (10–30 kV), and extrusion rate (0.6–1.2 mL/h). The surface density of amino group on the PVA–chitosan nanofiber membrane was determined based on the binding capacity of PVA–chitosan composite nanofiber for Acid Orange 7 (AO7) [22]. At pH 2, the amine functional groups (–NH_3_^+^) in PVA–chitosan nanofiber membrane are stoichiometrically equal to the sulfur trioxide functional groups (–SO_3_^−^) carried by AO7. 

#### 2.2.2. Preparation of HTCC

HTCC was prepared by coupling chitosan with GTMAC following the procedures reported elsewhere [34,35]. In brief, 1 g of chitosan (average *Mw*: 340 kD) was first dispersed in 100 mL of acetic acid solution to make up 1% (*w*/*v*) chitosan-dispersed solution. After transferring the mixture to a 3-neck round bottom flask (250 mL), 2.5 g of GTMAC was added. The mixture was subjected to heating for 24 h at 80 °C and 200 rpm. Next, any unreacted GTMAC was removed via the dialysis of solution. Later, the clear solution was concentrated under vacuum at 70 °C using a rotary evaporator. To induce the formation of precipitate, the concentrated solution was subsequently submerged in an ice bath of cold acetone (400 mL). The collected precipitates were then rinsed with cold acetone twice. After that, the precipitates were dried at 105 °C for 12 h. The resultant HTCC was used as the final product.

#### 2.2.3. Determination of Degree of Quaternization of HTCC

A titration method was applied for the determination of the degree of quaternization (DQ) of HTCC. Firstly, an appropriate amount of HTCC was dissolved in the deionized water. Potassium chromate was added to the solution as an indicator. The solution was titrated with the silver nitrate (AgNO_3_) solution until the first appearance of red–brown coloration was observed, indicating the complete reaction of all Cl^−^ ions in the titration process. The volume of titrant used was recorded, and the DQ of quaternary ammonium salt groups was evaluated according to Equation (1) [29,36,37]:

(1) $$DQ\left(\%\right)=\left(\frac{V\times C_{M}}{V\times C_{M}+\left(W_{HTCC}-V\times C_{M}\times 314\right)/161}\right)\times 100$$

where *W* is the weight of HTCC (g), *V* is the volume of titrant (mL), and *C_M_* (mol/L) is the titrant concentration (AgNO_3_ solution). The numbers 314 and 161 correspond to the molecular weights of the repeated structural units of HTCC and chitosan, respectively. 

#### 2.2.4. Preparation of PVA–HTCC Nanofiber Membrane 

**Figure 2:**
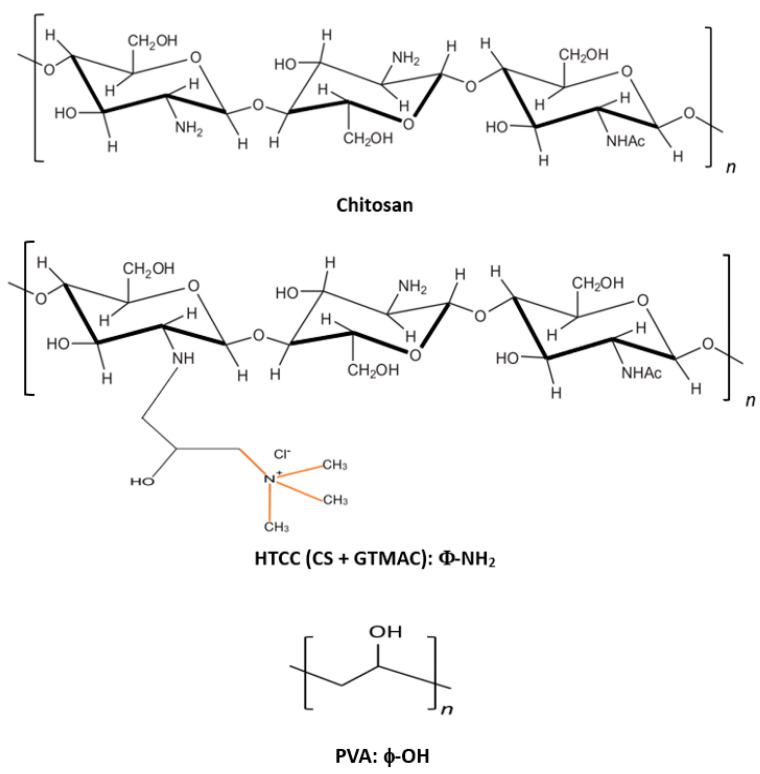
Chemical structures of chitosan (CS), HTCC, and PVA.

The stock solutions containing 10% (*w*/*w*) PVA and 3% (*w*/*w*) HTCC were prepared separately by dissolving HTCC and PVA in the distilled water at 25 °C and 80 °C, respectively. The two solutions were stirred at 200 rpm for 24 h. The electrospinning solutions were prepared by mixing the prepared HTCC and PVA stock solutions at different mass ratios at 200 rpm and 25 °C for 24 h. Before the electrospinning process, the stock solutions of PVA and HTCC as well as the PVA–HTCC solution must be well mixed and clear. The ranges of variables tested in the electrospinning experiments are listed as follows: PVA:HTCC ratio (90/10–50/50), applied voltage (15 kV), and extrusion rate (1.2 mL/h). Lastly, the resultant PVA–HTCC composite nanofiber membranes were subjected to a heat pressing process at 125 °C for 1 h. The chemical structures of PVA, chitosan, and HTCC are shown in Figure 2. In our previous study, FTIR spectra of PVA, chitosan, PVA–chitosan nanofiber membrane, and PVA–HTCC nanofiber membrane have been reported [29].

#### 2.2.5. Measuring the Diameter of the Nanofibers

A manual method is a commonly used method for measuring the diameter of nanofibers. First, a scale was set; then, pixels located between two edges of the vertical axis were counted. The number of pixels was converted to nanometer (nm) and the results of the nanofiber diameter was obtained. Depending on the resolution of an image, 30 to 100 measurements of the fiber diameter were made. The average nanofiber diameter was then further calculated.

### 2.3. Water Solubility Test

The PVA–HTCC nanofiber membranes were first cut into circular pieces with an area of 4.91 cm^2^ (or i.d. = 25 mm). Then, the circular pieces were dried at 60 °C for 24 h in a vacuum oven. The water solubility test was conducted by immersing the pieces of nanofiber membranes in 10 mL of water at 25 °C for 2 h. After incubation, the circles were first removed from water and then dried in an oven at 105 °C. The dried weight of the sample (*W*) was determined using Equation (2):

(2) $$W=\frac{W_{i}-W_{f}}{W_{i}}\times 100\%$$

where *W_i_* and *W_f_* are the initial and final weights of the same piece of nanofiber membrane, respectively. All the experiments were performed in triplicate and the average values are reported. The percentage of water solubility of PVA–HTCC can be correlated by the Monod-type model shown in Equation (3) [38]:

(3) $$W_{s}=\frac{W_{max}\cdot t}{K_{R}+t}$$

where *W_s_* is the percentage of water solubility of PVA–HTCC nanofiber membrane (%) at time *t* (h), *W_max_* is the maximum percentage of water solubility (%), and *K_R_* is the time required for the solubility of the PVA–HTCC nanofiber membrane to become half its maximum value (h). The maximum percentage of water solubility can be calculated from the slope and intercept of the plot of a linearized Monod-type Equation (4):

(4) $$\frac{t}{W_{s}}==\frac{K_{R}}{W_{max}}+\frac{t}{W_{max}}$$

### 2.4. Antibacterial Analysis 

The antibacterial property of nanofiber membranes was analyzed using the AATCC 100 test method with slight modifications [39,40]. The detailed procedure of antibacterial test was described in a previous study [33]. *E. coli* was cultured in Luria-Bertani (LB) liquid broth at 37 °C for 24 h. A cell suspension was then prepared at ~2 × 10^7^ colony-forming unit (CFU)/mL. The pieces of PVA-HTCC nanofiber membranes (circular shape with inner diameter of 25 mm; area = 4.91 cm^2^; weight = ~0.03 g) were incubated in 200 μL of *E. coli* cell suspension (~2 × 10^7^ cells/mL) for 24 h. Then, the nanofiber membrane was washed with 20 mL of saline solution (0.85%). The antibacterial efficacy (*AE*) of the nanofiber membrane was determined using Equation (5) listed below:

(5) $$AE=\frac{A-B}{A}\times 100\%$$

where *A* indicates the number of CFU formed on the culture broth that had no contact with the nanofiber membrane, while *B* is the number of CFU formed on the culture broth in which the treated nanofiber membranes were immersed for 24 h. Experiments were conducted in duplicate, and the average values of triplicate readings of CFU are reported. 

The regeneration of the PVA–HTCC nanofiber membrane after the antibacterial analysis was performed by incubating the membrane in 20 mL of 0.85% NaCl solution at 200 rpm, and 4 °C for 5 min. The regenerated nanofiber membranes were subjected to repeated uses, and their antibacterial performance was analyzed in terms of *AE* values.

### 2.5. Assessment of Cytotoxicity of Nanofiber Membranes

The procedures of accessing the cytotoxicity of the PVA-HTCC nanofiber membrane was based on an indirect method (i.e., assessment of cell viability) reported elsewhere [29]. Cell viability (i.e., cell survival rate) was qualitatively confirmed using live/dead cell fluorescent imaging [29]. To prepare the sample extract solution, a sheet of PVA–HTCC nanofiber membrane (diameter: 25 mm, surface area: 4.9 cm^2^) was firstly soaked in Dulbecco’s Modified Eagle Medium (DMEM) (volume = 1 mL) for 24 h of treatment. Simultaneously, mouse fibroblasts L929 cells were seeded in the wells of a 96-well plate and were incubated in a humidified 5 % CO_2_ atmosphere at 37 °C for 24 h. Then, the sample extract solution (100 μL) was loaded into the wells containing L929 cells at concentration = 1 × 10^4^ cells/well. After 3 days of incubation, 10 μL of CCK-8 solution was added to each well, and the 96-well plate was continuously incubated at 37 °C for 1 h, followed by the measurement of OD value at wavelength 450 nm using a microplate reader (SpectraMax M5, Molecular Devices, NY, USA). Each experiment was performed in triplicate and the results obtained were expressed as mean ± standard error. Statistical analysis was performed, and a *p* value less than 0.05 indicates statistical significance.

## 3. Results and Discussion

### 3.1. FTIR Spectra of Nanofiber Membranes

**Figure 3:**
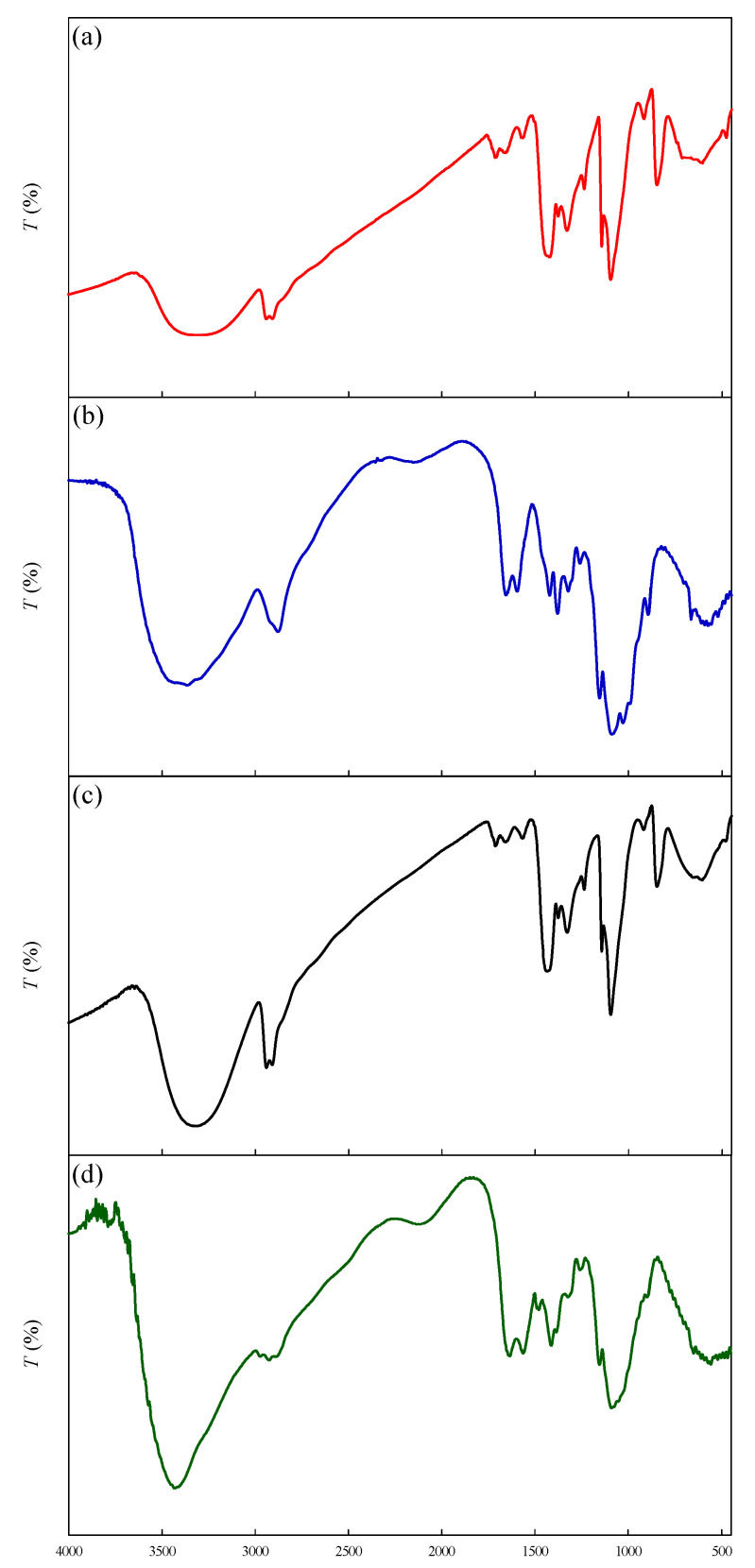
FTIR spectra of (**a**) PVA, (**b**) chitosan, (**c**) PVA–chitosan nanofiber membrane, and (**d**) PVA–HTCC nanofiber membrane.

Figure 3a–d show the FTIR spectra of PVA, chitosan, PVA–chitosan nanofiber membrane, and PVA–HTCC nanofiber membrane. Similar results have been shown by Wu et al. (2021) [29]. The peaks shown in FTIR spectra of PVA [Figure 3a] were similar to that of PVA–chitosan [Figure 3c] nanofiber membrane, except that the absorption peak at 1260 cm^−1^ (O-H) was absent in the FTIR spectrum of PVA–chitosan nanofiber membrane. The region of 3000–3600 cm^−1^ in the FTIR spectra corresponds to the O-H stretching vibration of the hydroxyl group. The bands appearing at around 1650 and 1570 cm^−1^ indicate the presence of amino group in chitosan [5]. 

As shown in Figure 3b for chitosan and Figure 3d for PVA–HTCC, the N-H stretching of the primary amino group in chitosan was proven by a resonance peak emerging at 3429 cm^−1^ in the FTIR spectrum [23], which confirms the successful synthesis of HTCC. Moreover, HTCC’s FTIR spectrum is associated with other significant absorption bands: (1) the –OH and –NH stretching vibration, emerging at ~3300 and ~3200 cm^−1^, respectively, (2) the N^+^-H stretching vibration, appearing at ~3037 cm^−1^, and (3) the CH_2_ vibration, emerging at ~2993 and ~2845 cm^−1^. Additionally, the strong peak present at ~1481 cm^−1^ suggests the presence of C-H bending of methyl substituent of the quaternary ammonium groups. The absorption bands emerging at ~1646 and ~1357 cm^−1^ indicate the existence of the C=O and C-O stretching of amide groups, respectively. The occurrence of the C-O-C antisymmetric stretching (at ~1167 cm^−1^), skeletal vibration involving C-O stretching (~1020 cm^−1^), and skeletal vibration involving C-C stretching (~970–850 cm^−1^) in Figure 3d confirmed the presence of polysaccharide skeleton [40].

### 3.2. Degree of Quaternization of HTCC

Equation (1) was used to estimate the DQ value of the quaternary ammonium salt groups [36]. The hydrogen atoms of –NH_2_ group in the chitosan molecule can be replaced by the group of a quaternary ammonium salt. Furthermore, the substituted quaternary ammonium salt group was expressed using the DQ value. In this work, the DQ value of the synthesized HTCC was 77.6 ± 3.7% by Equation (1). This DQ value approached the value (DQ 76.4 ± 4.3%) reported by Xue et al. (2014) [24]. The results confirmed that the substitution of amino groups by the quaternary ammonium salt groups in chitosan was present. The DQ value may affect the charge density on the PVA–HTCC nanofiber membrane. The degree of charge density may further affect the possibility of electro-spinnability of PVA–HTCC solution and the antibacterial efficiency of the resultant nanofiber membrane. The mass ratios of PVA to chitosan or HTCC in PVA–chitosan and PVA–HTCC nanofiber membranes were set at 90:10, and the corresponding weights of nanofiber membranes were approximately ~0.0135 g and ~0.0015 g.

### 3.3. Optimization Process of PVA–Chitosan Nanofiber Membranes

**Figure 4:**
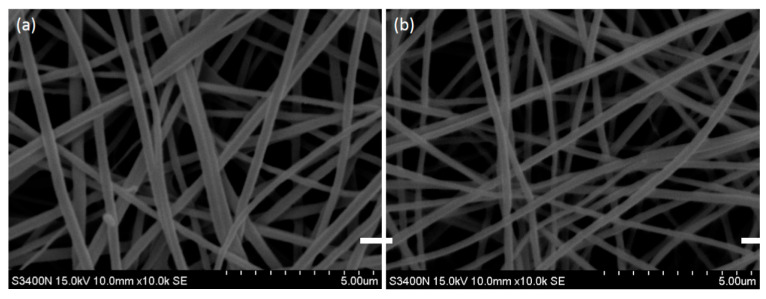

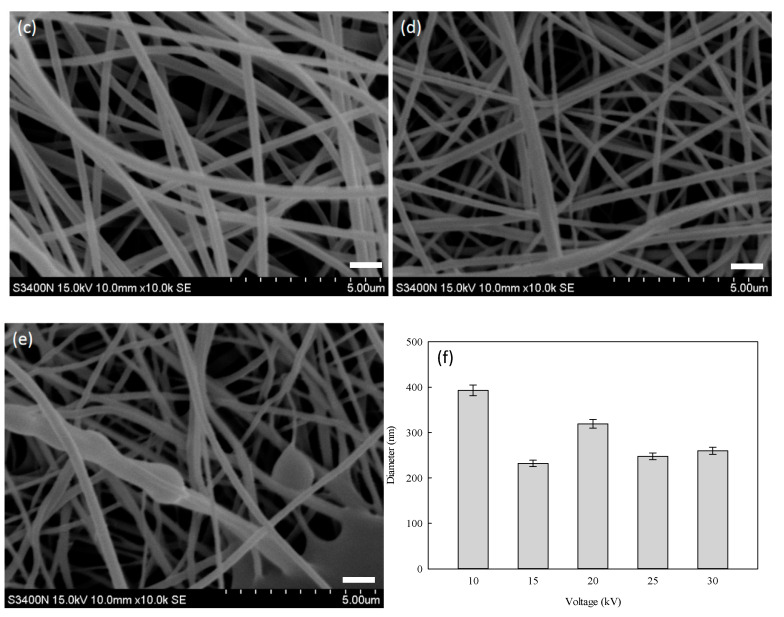
SEM images of PVA–chitosan nanofiber membranes prepared at (**a**) 10 kV, (**b**) 15 kV, (**c**) 20 kV, (**d**) 25 kV, and (**e**) 30 kV, using a constant mass ratio of PVA to chitosan at 9:1. (**f**) Average diameter of nanofibers (nm) as a function of the applied voltage (kV). Distance: 15.8 cm; extrusion rate: 1.2 mL/h. Scale bar: 1000 nm.

The morphological properties of the PVA–chitosan nanofibers fabricated using different electrospinning conditions were evaluated systematically. The effect of electrospinning voltage on the properties of nanofiber was evaluated by varying the voltage in the range of 10–30 kV, while keeping the following parameters at constant: PVA-to-chitosan mass ratio (9:1), concentrations of PVA (10%) and chitosan (3%), extrusion rate (1.2 mL/h), and spinning distance (15.8 cm). As shown in Figure 4a–e, the average fiber diameter reduced from 330 nm to 240 nm when the electrospinning voltage increased from 10 kV to 25 kV. The electrospun nanofibers at 10, 15, and 20 kV were more uniform in diameter (average fiber diameter: 330 nm for 10 kV, 250 nm for 15 kV, and 320 nm for 20 kV). An increase in the applied voltage beyond the critical value results in the formation of beaded nanofibers. The beaded nanofibers formed at a higher applied voltage were attributed to the decrease in the size of the Taylor cone and the increase in the jet velocity at the same flow rate [41]. A larger size distribution and some bead-like structures were observed when the voltage increased to 30 kV. Nonetheless, the nanofiber diameter did not change significantly with the increasing voltage, as shown in Figure 4f. The results showed that the nanofiber diameter obtained from 15 kV was smaller than that obtained from 10 kV or 20 kV. In this work, 15 kV of operating voltage was chosen for the electrospinning of the PVA-chitosan solution. 

**Figure 5:**
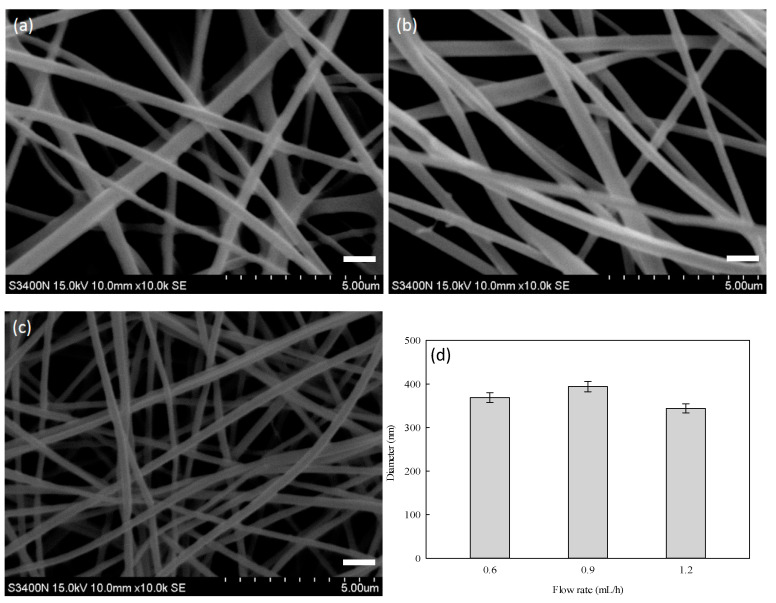
SEM images of PVA–chitosan nanofiber membranes prepared at (**a**) 0.6 mL/h, (**b**) 0.9 mL/h, and (**c**) 1.2 mL/h, using a constant mass ratio of PVA to chitosan at 9:1. (**d**) Average diameter of nanofibers (nm) as a function of the extrusion rate (mL/h). Distance: 15.8 cm; voltage: 15 kV. Scale bar: 1000 nm.

Next, the effect of extrusion rate on the morphology of nanofibers was investigated. The experiment was carried out by varying the extrusion rate between 0.6 and 1.2 mL/h; SEM images of the resultant nanofibers are shown in Figure 5a–c. The structure of nanofiber became denser (i.e., number of fibers per unit area) as the extrusion rate increased. Moreover, an increase in the roughness of the fiber surface was observed when a lower extrusion rate (0.6 and 0.9 mL/h) was applied; this phenomenon may be caused by the insufficient amount of the polymer blend needed for electrospinning process. The surface of the nanofiber was found to be smoother when the extrusion rate was increased to 1.2 mL/h. This indicates the improvement in electrospinning stability. The diameters of nanofibers electrospun at 0.6 and 0.9 mL/h were 400 nm and 440 nm, respectively. For nanofibers electrospun at 1.2 mL/h, the diameter and the size distribution were smaller, as shown in Figure 5d. Therefore, 1.2 mL/h was chosen as the optimal extrusion rate for the next batch of nanofiber electrospinning study.

**Figure 6:**
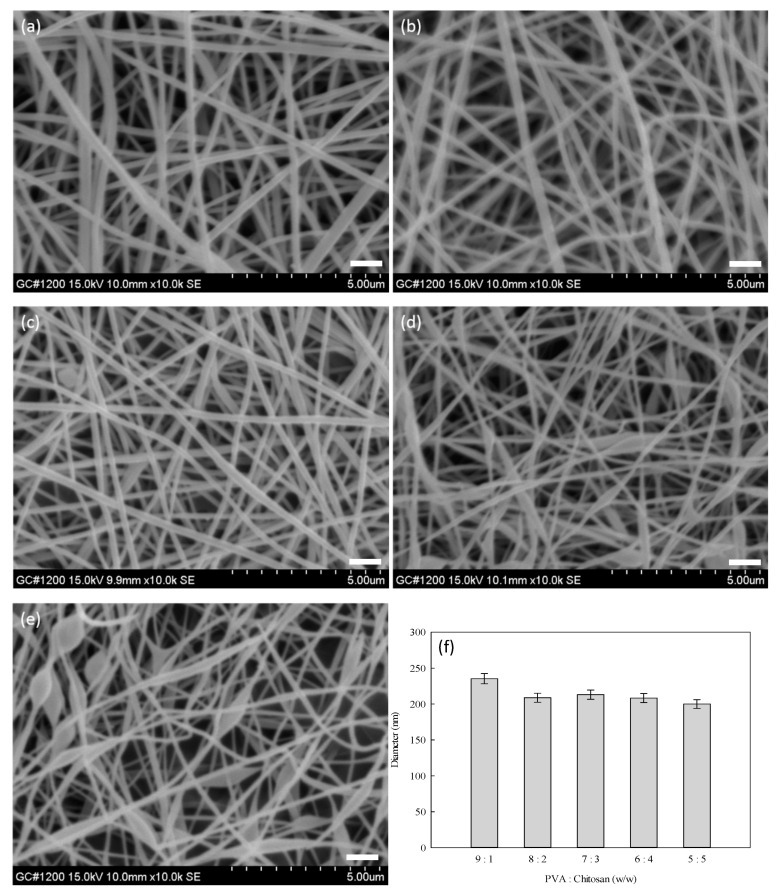
SEM images of PVA–chitosan nanofiber membranes prepared using mass ratios of PVA to chitosan at (**a**) 9:1, (**b**) 8:2, (**c**) 7:3, (**d**) 6:4, and (**e**) 5:5. (**f**) Average diameter of nanofibers (nm) as a function of the mass ratio of PVA to chitosan. Distance: 15.8 cm; voltage: 15 kV; extrusion rate: 1.2 mL/h. Scale bar: 1000 nm.

It has been known that chitosan is a cationic polysaccharide with amino groups, which are ionizable under acidic conditions (i.e., pH > 6.5) [42]. Hence, the morphology and average diameter of electrospun nanofibers were influenced by the mass ratio of PVA–chitosan. Figure 6 shows the SEM images of PVA–chitosan nanofibers synthesized at different mass ratios of PVA to chitosan. As shown in Figure 6a–c, the sizes of the nanofiber membranes produced at the PVA-to-chitosan mass ratio ranging from 9:1 to 7:3 were relatively uniform. At 6:4 and 5:5 of the PVA:chitosan mass ratio, an irregular shape of the electrospun fibers was observed under the microscope, as shown in Figure 6d,e. On the other hand, when the chitosan content was more than 40%, the bead-like structure in the nanofiber mats was formed. This undesirable structure may be due to the repulsive force between ionic groups within the chitosan polymer backbone that was expected to inhibit the formation of continuous nanofiber during electrospinning; hence, an increase in the applied voltage beyond the critical value resulted in the formation of beaded nanofibers. Similar results were also observed by Haider et al. (2018) [41], Jia et al. (2007) [42], and Paipitak et al. (2011) [43].

As shown in Figure 6f, it was found that the average diameter of the nanofibers decreased with increasing chitosan content in the mixed solution. The nanofiber prepared at a 9:1 mass ratio of PVA to chitosan was found to be the most uniform. This observation could be explained by the fact that a higher concentration of chitosan with a higher amount of ionic group promoted the charge density on the jet surface. The antibacterial efficiency of nanofiber membranes prepared at different mass ratios of PVA, and chitosan was investigated in the subsequent study.

### 3.4. Optimization Process of PVA–HTCC Nanofiber Membrane

**Figure 7:**
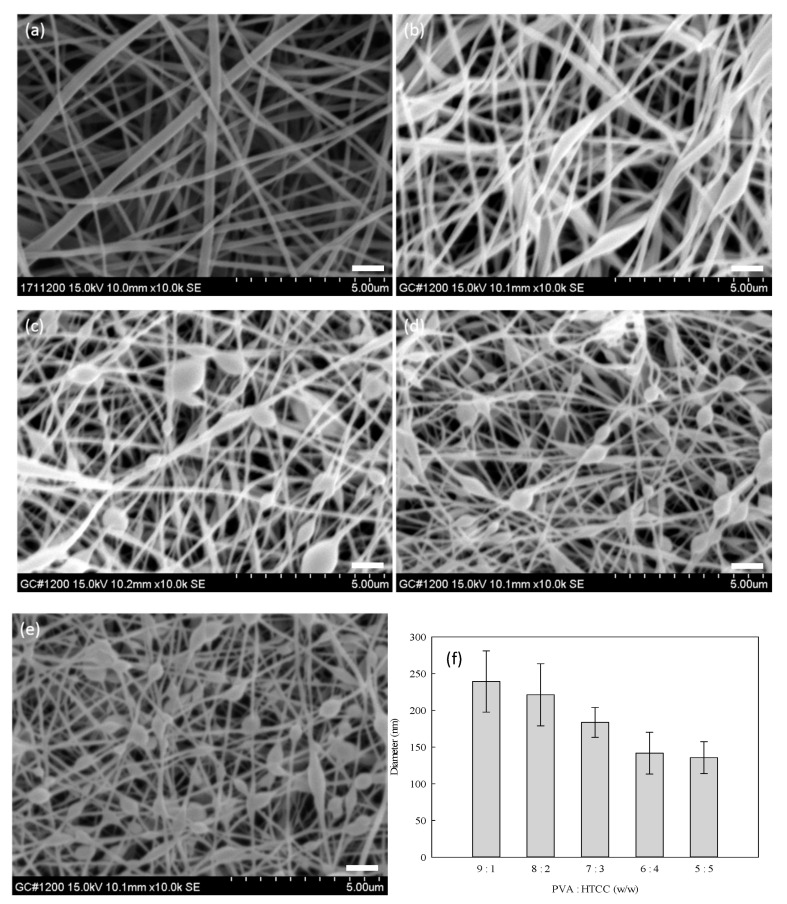
SEM images of PVA–HTCC nanofiber membranes prepared using mass ratios of PVA to HTCC at (**a**) 9:1, (**b**) 8:2, (**c**) 7:3, (**d**) 6:4, and (**e**) 5:5. (**f**) Average diameter of nanofibers (nm) as a function of the mass ratio of PVA to HTCC. Distance: 15.8 cm; voltage: 15 kV; extrusion rate: 1.2 mL/h. Scale bar: 1000 nm.

The PVA–HTCC nanofiber membranes were prepared using different concentrations of HTCC (10–50%) in the PVA–HTCC mixture. The SEM images of PVA–HTCC nanofibers are illustrated in Figure 7a–e. The composition of electrospinning solution influenced the morphology, average diameter, and size distribution of the nanofibers. As given in Figure 7f, the diameters of PVA–HTCC nanofibers ranges from 239 nm to 135 nm, corresponding to the ranges of PVA:HTCC mass ratio used (from 9:1 to 5:5). The average diameter of nanofibers fabricated from PVA:HTCC (90:10) was larger than that of the fibers fabricated from the solutions containing a higher concentration of HTCC, as shown in Figure 7a. This is due to the reduction in the electrospinning ability when a higher concentration of water-soluble HTCC in the mixed solution was used. In addition, a bead-like structure was observed in the nanofiber mats when the concentration of HTCC increased from 20% to 50% [see Figure 7b–e], and the average diameter of spun-nanofibers decreased when HTCC content increased in the mixed solution. Similar results were also observed by Deng et al. (2012) [25]. The bead structure was not formed when a lower concentration of HTCC (10%) was used, as shown in Figure 7a. Hence, a mass ratio of PVA to HTCC at 9:1 was used in the subsequent studies. 

### 3.5. Water Solubility of PVA–HTCC Nanofiber Membranes

**Figure 8:**
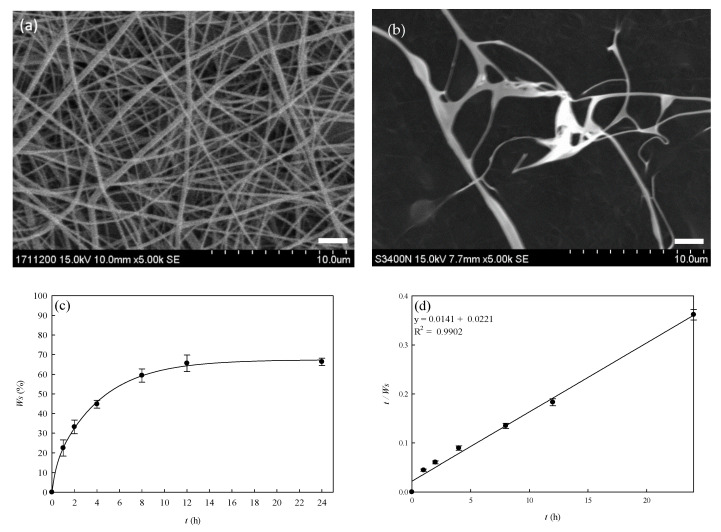
SEM photos of heat-pressed PVA–HTCC nanofiber membranes in (**a**) dry condition, and (**b**) after 2 h of immersion in water at 25 °C. (**c**) Water solubility of heat-pressed PVA–HTCC nanofiber membranes after 24 h of water incubation. (**d**) plot of *t/Ws* against *t* for assessing the maximum water solubility of PVA–HTCC nanofiber membrane. Scale bar: 2000 nm.

SEM images of the crosslinked PVA–HTCC nanofibers before and after the water immersion test are illustrated in Figure 8a,b. The porosity of the PVA–HTCC nanofiber membrane was found to be 83.67 (%). Since both PVA and HTCC are soluble in water, the structure of PVA–HTCC nanofiber membranes could collapse upon contact with water [44]. Hence, it was crucial to perform the water solubility test on the PVA–HTCC nanofiber membrane. It was found that the PVA–HTCC nanofiber membrane began to dissolve once it was exposed to water. As indicated in the plot of water solubility against time [Figure 8c], a 33.2% of weight loss was recorded after 2 h of water immersion. After 8 h of water immersion, the weight loss of the nanofiber had increased to 59.4%, and the weight of the nanofiber did not change prominently thereafter. The total weight loss of the nanofiber after contacting with water for 24 h was found to be ~66.3%, indicating that a large portion of nanofiber structure was dissolved in water.

Figure 8c shows the percentage of water solubility of PVA–HTCC nanofiber membrane in water at 25 °C over 24 h. The maximum water solubility (%) can be calculated using Equation (4). The slope and intercept of the plot are shown in Figure 8d. The maximum water solubility of PVA–HTCC nanofiber was ~70.92 (%) and the time required for 50% water solubility was ~0.639 h. The weight loss in the PVA–HTCC composite nanofiber in water was mainly due to the dissolution of HTCC. Based on Figure 8c, the water solubility of the PVA–HTCC nanofibers reached the maximum at 12 h. 

### 3.6. Antibacterial Performances of Nanofiber Membranes

#### 3.6.1. PVA–Chitosan Nanofiber Membrane

**Figure 9:**
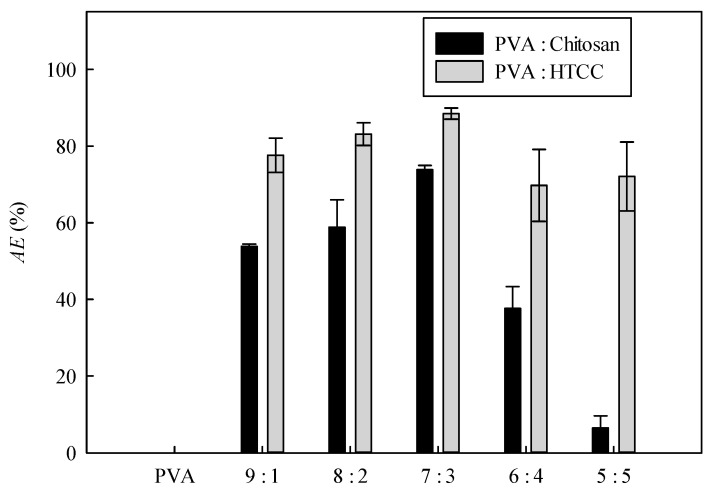
*AE* values of PVA–chitosan and PVA–HTCC nanofiber membranes prepared using a mass ratio of PVA to chitosan or HTCC at 9:1.

The *AE* values of PVA–chitosan nanofiber membranes synthesized at different PVA:chitosan ratios are shown in Figure 9. The order of PVA:chitosan mass ratios based on the descending *AE* values of PVA-chitosan nanofiber membranes is given as 7:3 > 8:2 > 9:1 > 6:4 > 5:5. The antibacterial effect is due to the positively charged amino groups interacting with a negatively charged *E. coli*, causing the microorganisms to release the proteins and other intracellular components [45]. In terms of antibacterial performance, the PVA–chitosan nanofiber membrane synthesized at mass ratio of 7:3 gave the highest *AE*, and the disinfection rate reached 73.82%. Nevertheless, the *AE* of the nanofiber membrane containing the highest chitosan content (PVA:chitosan mass ratio at 5:5) was only 6.47%. When chitosan molecules were present at a higher concentration, the *AE* of the PVA–chitosan nanofiber membrane increased as well. However, *AE* became lower when the chitosan content was as high as 50%; this might be due to the saturation of chitosan on the membrane surface, causing a less effective contact of chitosan with the *E. coli*. 

#### 3.6.2. Antibacterial Efficacy of PVA–HTCC Nanofiber Membrane

The quaternary ammonium group of HTCC enhances the chargeability and the antibacterial effect of nanofibers. The *AE* values of PVA–HTCC nanofiber membranes prepared at different mass ratios of PVA to HTCC (9:1 to 5:5) were determined. Figure 9 shows the antibacterial performances of PVA–HTCC nanofiber membranes. The PVA nanofiber membrane without HTCC or chitosan did not exhibit any antibacterial effect.

**Figure 10:**
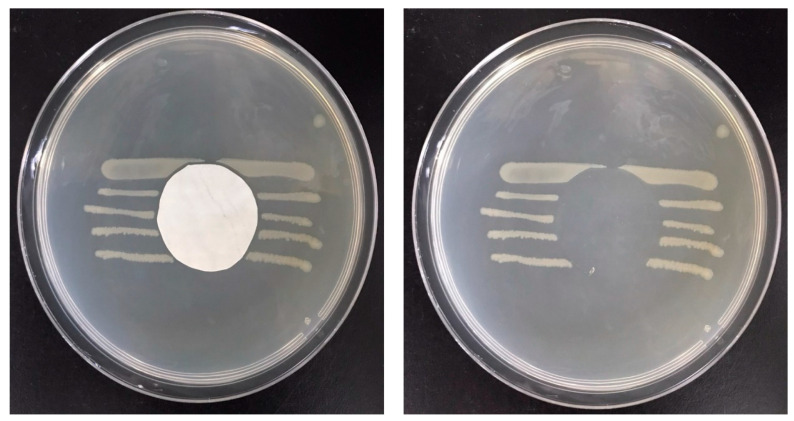
Images of *E. coli* streak culture after incubation with PVA–HTCC nanofiber membrane.

It can be observed that the *AE* of the PVA–HTCC nanofiber membrane was significantly higher than that of the PVA–chitosan nanofiber membrane prepared using the same mass ratio of PVA to chitosan or HTCC at 9:1. The result suggested that the quaternary ammonium salt of HTCC plays a vital role in the antibacterial action. As the mass ratio of PVA to HTCC changed from 9:1 to 7:3, the *AE* of the PVA–HTCC nanofiber membranes increased. Moreover, a decrease in the nanofiber diameter of PVA–HTCC nanofiber membranes was observed in the nanofiber membranes prepared using a higher PVA-to-HTCC mass ratio. The maximal *AE* (~90%) was attained using the PVA–HTCC nanofiber membrane prepared at a 7:3 mass ratio. It was postulated that an increase in the HTCC content leads to a higher positive charge density of the PVA–HTCC nanofiber, which may result in a higher degree of cell attachment to the nanofiber membrane. Similarly, when the PVA–HTCC fiber diameter increased, the amount of positively charged HTCC per unit area of the nanofiber membrane was lesser; hence, the number of *E. coli* cell attached to the nanofiber membrane was less. The antibacterial performance of the PVA–HTCC (mass ratio 7:3) membrane was also evaluated based on the growth of *E. coli* after the antibacterial treatment. Figure 10 illustrates the bacterial culture in agar plates treated by the PVA–HTCC nanofiber membrane. The antibacterial zone was not observed in this case. The results showed that the HTCC would not be released from the PVA–HTCC nanofiber membrane.

The antibacterial activity of HTCC was mainly attributed to the interactions between the positively charged quaternized amino groups in HTCC and the negatively charged microbial cell membrane, in which membrane permeability was negatively affected, allowing microbial proteins and other intracellular components to flow out; these interactions lead to the cell death [17]. The effective concentration of the quaternized amino group in the membrane structure increased as the HTCC concentration increased; this may improve antibacterial performance. At the mass ratios of 6:4 and 5:5, the *AE* value of PVA–HTCC nanofiber membranes decreased as the positive charge density of HTCC was too high for the *E. coli* to be difficult to attach to the surface of the composite membrane. This resulted in a decrease in antibacterial efficiency. Moreover, PVA–chitosan nanofiber membrane contains the positively charged amino groups (-NH_3_^+^, from chitosan) that can interact with the negatively charged membrane of a microbial cell. Hence, similar antibacterial behavior was noted for the PVA–chitosan nanofiber membrane. This phenomenon had a much higher impact on the antibacterial efficiency of the PVA–chitosan nanofiber membrane, as shown in Figure 9.

The reusability of PVA–HTCC nanofiber membranes was tested by five rounds of antibacterial treatments. After the five rounds of antibacterial treatments, the *AE* of PVA-HTCC nanofiber membranes remained at ~90%. The results suggested the high chemical stability of the fabricated PVA–HTCC (7:3) nanofiber membrane during the repeated uses; the charge density of the HTCC on the nanofiber membrane was well maintained, resulting in the similar antibacterial effect. Moreover, the PVA–HTCC nanofiber membrane maintained the same *AE* after a storage period of one month in dry state. 

#### 3.6.3. Cytotoxicity of Nanofiber Membranes

**Figure 11:**
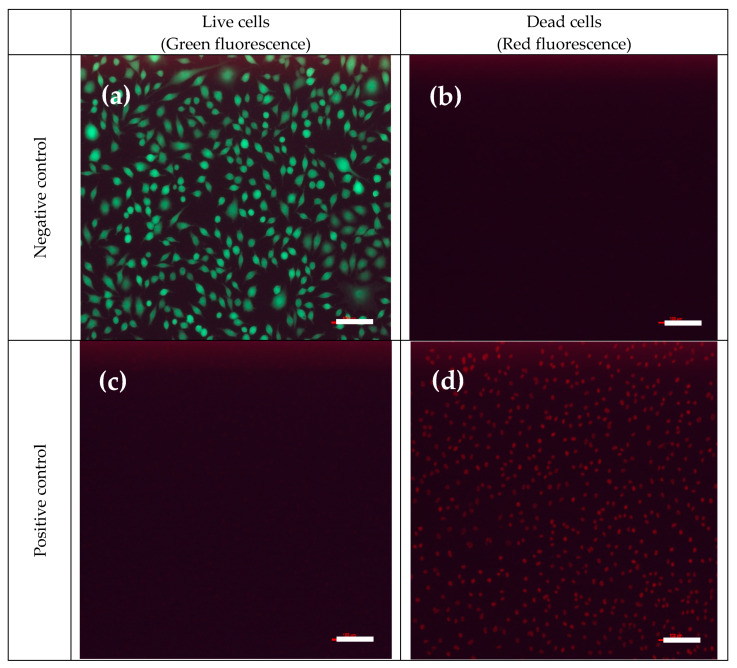

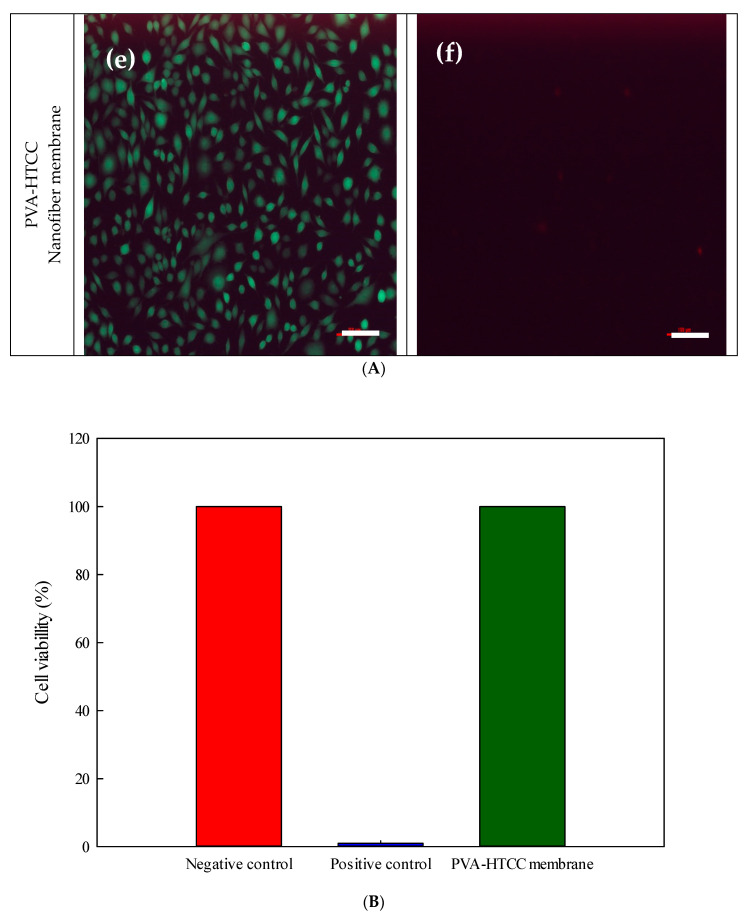
(**A**). Fluorescence images of live (green) and dead (red) cells. (**a**,**b**): Negative control; (**c**,**d**): Positive control; (**e**,**f**): PVA–HTCC nanofiber membrane sample. Scale bar: 100 μm. (**B**). Cytotoxicity tests (indirect tests) on mouse fibroblasts L929 using CCK-8 assay and samples. Results are presented as the mean value of three independent experiments with three replicates each.

The CCK-8 assay [29] was adopted in this study to evaluate the cytotoxicity of PVA–HTCC nanofiber membranes on mouse fibroblasts L929 cells. Cell viability (i.e., cell survival rate) was qualitatively confirmed using live/dead cell fluorescent imaging [29]. The cell images obtained from negative and positive controls are shown in Figure 11A(a–f). The negative control (i.e., ~100% of cell survival) was indicated by the green fluorescence signal [Figure 11A(a,b)], while the positive control (i.e., ~0% of cell survival) was indicated by the red fluorescence signal [Figure 11A(c,d)]. As shown in the fluorescence images, the PVA–HTCC nanofiber membrane did not show the cytotoxic effect on L929 cells [Figure 11A(e,f)]. The cytotoxicity of PVA/HTCC/BI nanofiber membrane was further quantitatively measured by indirect methods using CCK-8 assay. The cell viability for PVA–HTCC nanofiber membrane as analyzed using CCK-8 assay is shown in Figure 11B. The results proved the non-cytotoxicity of PVA–HTCC nanofiber membrane, as indicated by the ~100% of cell viability. Hence, this work demonstrated that the PVA–HTCC nanofiber membrane has good cell biocompatibility and shows no cytotoxicity to L929 cells. Hence, it also can be potentially utilized in biomedical applications.

## 4. Conclusions

The PVA–chitosan and PVA–HTCC nanofiber membranes were successfully prepared via electrospinning. The effects of blend ratio, applied voltage, and extrusion rate on the morphology of synthesized nanofiber membranes were investigated. Our results showed that the PVA–HTCC nanofibers exhibited slow and long-lasting release capacity in water, which provided high antibacterial activity against Gram-negative *E. coli*. Therefore, this type of composite nanofiber membrane can be used as a release-type antibacterial material. Moreover, it can be stored for a minimum of one month in a dry state and maintain the same antibacterial efficiency. The electrospun PVA–HTCC nanofiber membrane showed a high antibacterial activity against *E. coli*, but no cytotoxic effect to mouse fibroblast L929. The features reported in this study suggested that the PVA–HTCC nanofiber membrane can be a promising material for biomedical applications such as wound healing and drug delivery.

## Data Availability

Due to the nature of this research, participants in this study did not agree for their data to be shared publicly, so supporting data are not available.
